# Redefining germline predisposition in children with molecularly characterized ependymoma: a population-based 20-year cohort

**DOI:** 10.1186/s40478-022-01429-1

**Published:** 2022-08-25

**Authors:** Jon Foss-Skiftesvik, Ulrik Kristoffer Stoltze, Thomas van Overeem Hansen, Lise Barlebo Ahlborn, Erik Sørensen, Sisse Rye Ostrowski, Solvej Margrete Aldringer Kullegaard, Adrian Otamendi Laspiur, Linea Cecilie Melchior, David Scheie, Bjarne Winther Kristensen, Jane Skjøth-Rasmussen, Kjeld Schmiegelow, Karin Wadt, René Mathiasen

**Affiliations:** 1 Department of Pediatrics and Adolescent Medicine, Rigshospitalet University Hospital, Copenhagen, Denmark; 2 Department of Neurosurgery, Rigshospitalet University Hospital, Copenhagen, Denmark; 3 Department of Clinical Genetics, University of Copenhagen, Copenhagen, Denmark; 4 Department of Clinical Medicine, Faculty of Health and Medical Sciences, University of Copenhagen, Copenhagen, Denmark; 5 Department of Genomic Medicine, Rigshospitalet University Hospital, Copenhagen, Denmark; 6 Department of Clinical Immunology, Rigshospitalet University Hospital, Copenhagen, Denmark; 7 Department of Pathology, Rigshospitalet University Hospital, Copenhagen, Denmark; 8 Biotech Research and Innovation Center, University of Copenhagen, Copenhagen, Denmark; 9 Department of Health Technology, Cancer Systems Biology and Bioinformatics, Technical University of Denmark, Lyngby, Denmark; 10 Department of Neurosurgery, Section 6031, Rigshospitalet University Hospital, Inge Lehmanns Vej 6, 2100 Copenhagen, Denmark; 11 The Pediatric Oncology Research Laboratory, Section 5704, Department of Pediatrics and Adolescent Medicine, Rigshospitalet University Hospital, Henrik Harpestrengs Vej 6A, 2100 Copenhagen, Denmark

**Keywords:** DNA methylation profiling, Molecular classification, Genomics, Genetic susceptibility, Pediatrics

## Abstract

Ependymoma is the second most common malignant brain tumor in children. The etiology is largely unknown and germline DNA sequencing studies focusing on childhood ependymoma are limited. We therefore performed germline whole-genome sequencing on a population-based cohort of children diagnosed with ependymoma in Denmark over the past 20 years (n = 43). Single nucleotide and structural germline variants in 457 cancer related genes and 2986 highly evolutionarily constrained genes were assessed in 37 children with normal tissue available for sequencing. Molecular ependymoma classification was performed using DNA methylation profiling for 39 children with available tumor tissue. Pathogenic germline variants in known cancer predisposition genes were detected in 11% (4/37; *NF2*, *LZTR1*, *NF1* & *TP53*). However, DNA methylation profiling resulted in revision of the histopathological ependymoma diagnosis to non-ependymoma tumor types in 8% (3/39). This included the two children with pathogenic germline variants in *TP53* and *NF1* whose tumors were reclassified to a diffuse midline glioma and a rosette-forming glioneuronal tumor, respectively. Consequently, 50% (2/4) of children with pathogenic germline variants in fact had other tumor types. A meta-analysis combining our findings with pediatric pan-cancer germline sequencing studies showed an overall frequency of pathogenic germline variants of 3.4% (7/207) in children with ependymoma. In summary, less than 4% of childhood ependymoma is explained by genetic predisposition, virtually restricted to pathogenic variants in *NF2* and *NF1*. For children with other cancer predisposition syndromes, diagnostic reconsideration is recommended for ependymomas without molecular classification. Additionally, *LZTR1* is suggested as a novel putative ependymoma predisposition gene.

**Supplementary Information:**

The online version contains supplementary material available at 10.1186/s40478-022-01429-1.

## Introduction

Ependymoma is the second most common malignant central nervous system (CNS) tumor in children and is associated with poor long-term survival [1, 2]. Apart from a very limited number of children with neurofibromatosis type-2 associated spinal ependymoma, the underlying causes of ependymoma remain unknown [3, 4]. Several factors indicate that genetic predisposition plays a role including increased population-based familial risk [5], reports of familial intracranial ependymoma [6, 7], genetic ancestry-based risk differences [8] and an absence of known environmental risk factors [9].

No systematic germline sequencing investigation of genetic predisposition specific to childhood ependymoma has been reported to date. Over the last decade, several large pediatric pan-cancer germline sequencing studies have been performed, with childhood ependymoma accounting for less than 5% (191/4833) of the combined sample size [10–19]. Taken together, these whole-exome/-genome sequencing (WES/WGS) studies report rare pathogenic germline variants in 4.7% (9/191) of children with ependymoma, although individual study estimates range from 0 to 21%. Lack of molecular tumor classification [10, 11, 13–19], small ependymoma sample sizes [11, 12, 14–19], restriction to gene panels [10–19] and lack of population-based study designs [10–14, 16–19] further complicate the delineation of the nature and extent of genetic predisposition in childhood ependymoma.

The aim of this population-based study was to investigate genetic predisposition in children with molecularly classified ependymoma due to rare pathogenic germline variants both in and outside known cancer genes. Moreover, we assessed the feasibility of performing germline WGS and tumor DNA methylation profiling in a combined retro-/prospective nationwide cohort spanning more than 20 years.

## Material and methods

### Retrospective cohort

Children (< 18 years) diagnosed with ependymoma from 2000 to 2016 in Denmark were identified through the Danish Childhood Cancer Registry (DCCR) [20]. Registry data on date of birth, gender, histopathology and tumor location was validated by cross-linkage with the National Pathology Registry. Living patients aged > 18 years at the time of the study were informed and offered inclusion both in writing and by telephone. For minors (< 18 years at the time of the study) and for deceased patients, parents or legal guardians were contacted. Detailed clinical and four-generational pedigrees were retrieved through patient health record review for included patients.

### Prospective cohort

Since 2016, all children (< 18 years) diagnosed with cancer in Denmark have been offered germline WGS through the STAGING study, described in detail elsewhere [15, 21]. The prospective cohort consists of children with ependymoma included in STAGING from 2016 to 2021. Similarly to the retrospective cohort, data variables were retrieved through patient health record and histopathology report review.

### Collection of tissue for germline DNA sequencing

Leukocyte DNA was isolated from peripheral blood samples drawn in parallel with clinical sampling when possible. For deceased patients, archived blood samples were collected from the Copenhagen Hospital Biobank (CHB) [22]. For those without obtainable blood samples, dissection of normal brain tissue was performed on formalin-fixed paraffin embedded (FFPE) tumor tissue samples.

### Germline whole-genome and -exome sequencing

Germline WGS was performed on leukocyte DNA using the HiSeqX platform (Illumina, USA) with paired-end sequencing of 150 bp reads and target 30X average coverage. Germline WES of healthy brain tissue was performed using Novaseq 6000 (Illumina, USA). Exomes were sequenced as 2 × 150 bp paired-end reads to an average median coverage of 60X. Tissue handling, sequencing and bioinformatics procedures including variant filtering are further detailed in the Additional file 1: Methods.

### Cancer gene panel analysis

For the gene panel analysis, WGS/WES data was limited to filtered SNVs and SV deletions identified in a predefined set of 457 genes. This panel consisted of 390 cancer related genes supplemented by 67 genes with either established or suggested roles in ependymoma tumorigenesis selected based on the scientific literature (Additional file 2: Tables S1 and S2). Variants were reviewed by a multidisciplinary team specialized in pediatric cancer predisposition. Variants were classified as either “benign”, “likely benign”, “likely pathogenic”, “pathogenic”, or as “variants of unknown significance” (VUS) in accordance with international standards [23]. In the context of this study, “likely pathogenic” and “pathogenic” variants are referred to simply as “pathogenic”.

### Constrained gene analysis

For the constrained gene analysis, all rare, coding SNVs and SV deletions, were subsetted to variants predicted to cause loss-of-function (pLoF) in 2986 highly constrained genes. Based on metadata from 141,456 humans without serious childhood disease, evolutionarily constrained genes were defined by a LoF observed/expected upper bound fractions (LOEUF) score of ≤ 0.35 which is indicative of depletion of pLoF variation and in line with recent recommendations [24, 25]. Curation of resulting variants, including use of 586 in-house whole genome sequences from children with cancers other than ependymoma, is detailed in the Additional file 1: Methods and Additional file 2: Table S3.

### Tumor DNA methylation profiling and molecular classification

**Fig. 1:**
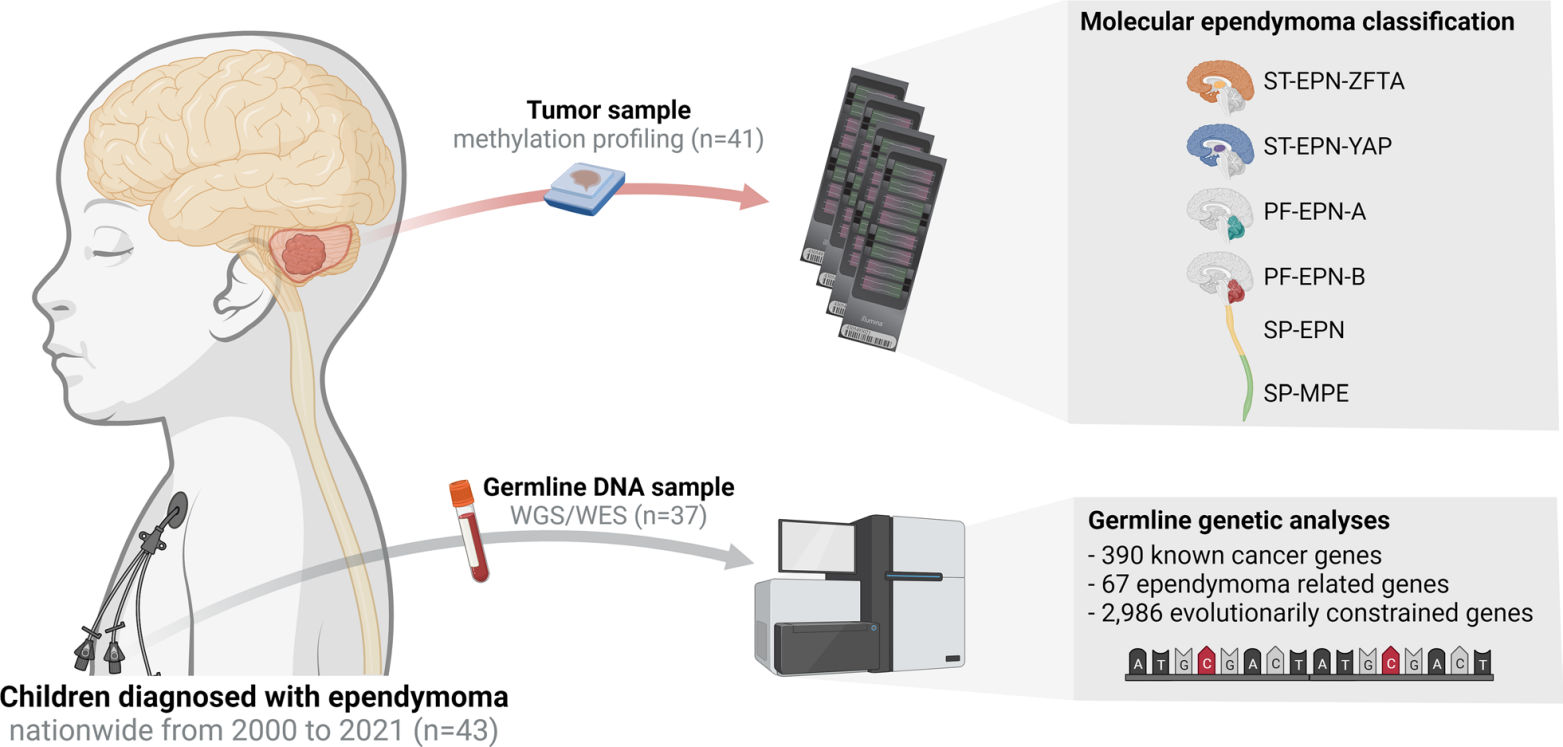
Graphic overview of the cohort (n = 43 children) and methods employed

Molecular tumor classification was performed using retrospectively collected iDAT files for patients with existing clinical DNA methylation profiles. For all others, archived FFPE or freshly frozen (FF) tumor samples were collected and underwent DNA methylation profiling using the Infinium MethylationEPIC BeadChip Kit (Illumina, USA). Archived tumor DNA was restored using the Infinium HD FFPE DNA Restore Kit (Illumina, USA) prior to methylation profiling. Tumor methylation class and subclass were predicted using a publicly available classifier tool [26]. The classifier version and employed cut-off scores are further detailed in Additional file 1: Methods. For an illustrative overview of the cohort and methods used, please see Fig. 1).

### Statistical analyses

Statistical analyses were performed using R v.3.6.1 and IBM SPSS Statistics v.25.

### Ethical approvals

This study was approved by the Capital Region Scientific Ethical Committee (H-15016782, prospective cohort) and the Danish National Committee on Health Research Ethics (2000407). All patients and/or parents/legal guardians provided informed consent.

## Results

### Patient characteristics

A total of 43 children registered with an ependymoma diagnosis were included. Median age at diagnosis (5.3, SD 4.7), gender distribution (females 44.2%), histopathology diagnosis, and tumor location (Table 1) were in line with existing population-based reports [27–29]. The overall inclusion rate was 77% (43/56). For the retrospective cohort, in which both living and deceased patients were eligible for inclusion, a higher rate of inclusion was seen for deceased patients compared to living patients (91% vs. 66%, Fisher’s exact test, *p* = 0.067). The inclusion process, including main reasons for exclusion, is illustrated in Additional file 1: Fig. S3.

**Table 1:** Patient clinical characteristics

Patient characteristics	n (% of total)
Total	43 (100%)
Median age at diagnosis, y (SD)	5.3 (4.7)
* Status*	
Alive	27 (62.8%)
Deceased	16 (37.2%)
* Gender*	
Female	19 (44.2%)
Male	24 (55.8%)
* Cohort*	
Retrospective	34 (79.1%)
Prospective	9 (20.9%)
* Histopathological diagnosis*	
Myxopapillary ependymoma, WHO 2	1 (2.3%)
Ependymoma, WHO 2	14 (32.6%)
Ependymoma, WHO 3	26 (60.5%)
Other*	2 (4.7%)
* Tumor location*	
Supratentorial	7 (16.3%)
Posterior fossa	30 (69.8%)
Spinal	5 (11.6%)
Multifocal**	1 (2.3%)

^*^Includes one patient initially diagnosed with atypical glioblastoma for whom subsequent clinical tumor methylation profiling resulted in an ependymoma diagnosis and one patient with ependymoblastoma incorrectly registered as ependymoma

^**^Includes one patient with disseminated ependymoma at diagnosis with tumor tissue located adherent to the insular cortex, the ventral surface of the brainstem and the caudal spinal cord

SD, standard deviation; y, years; WHO, the World Health Organization histological grade

### Molecular tumor classification

**Fig. 2:**
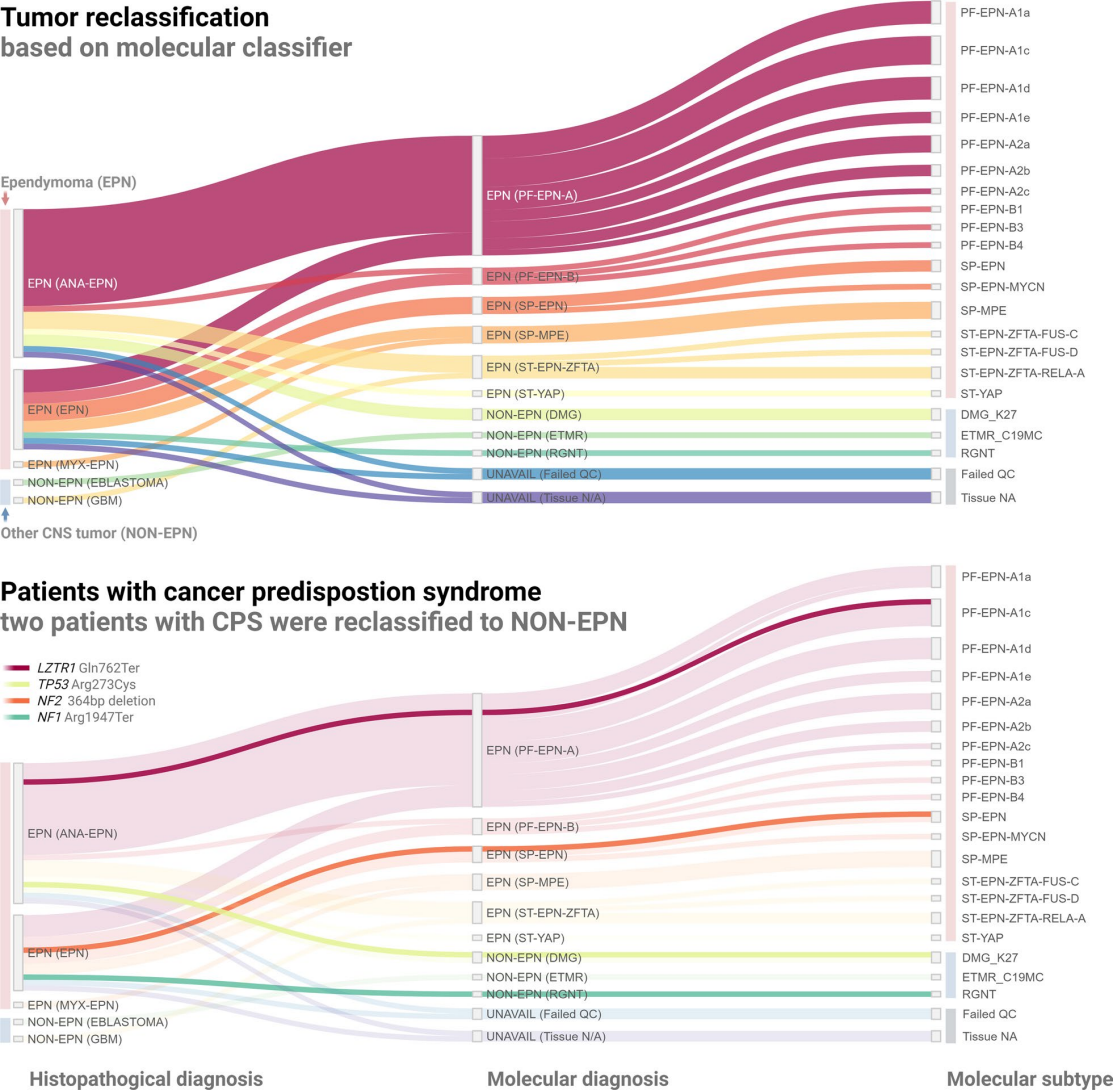
Sankey plot illustrating original histopathological diagnosis (left) for 43 children registered with ependymoma and corresponding tumor methylation class (right). CPS, cancer predisposition syndrome; ANA-EPN, ependymoma WHO 3; EPN, ependymoma WHO 2; MYX-EPN, myxopapillary ependymoma WHO 2; GBM, atypical glioblastoma with several differential diagnoses considered; where clinical methylation profiling resulted in alteration of the diagnosis; EBLASTOMA, ependymoblastoma incorrectly registered in the Danish Childhood Cancer Registry as ependymoma; NA, not available

Molecular tumor (re-)classification based on DNA methylation profiling was possible for 90% (39/43) of patients. Distribution of original histopathological diagnosis and resulting tumor methylation class and subclass is listed in Table 1 and illustrated in Fig. 2, respectively.

**Fig. 3:**
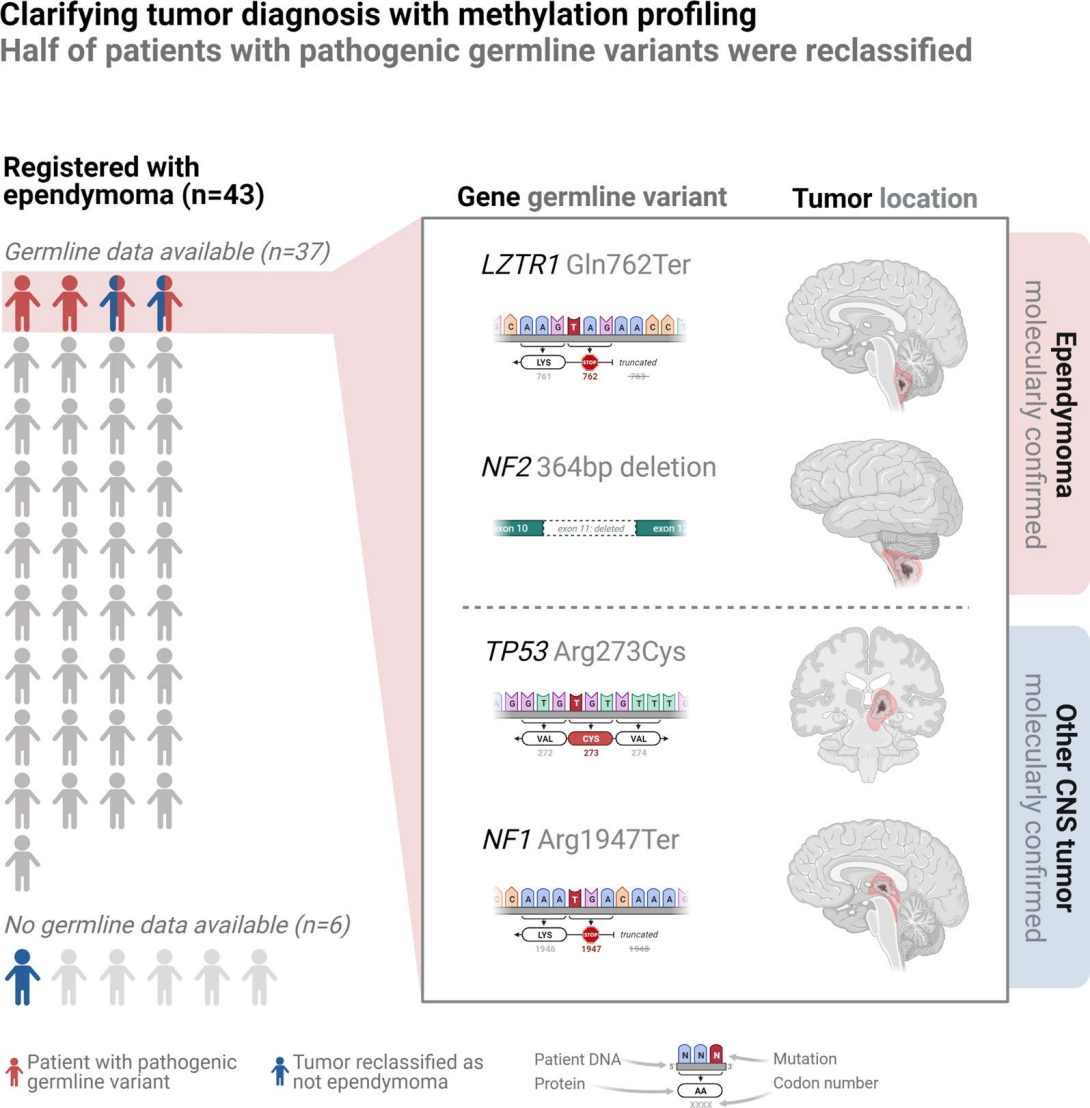
Overview of resulting molecular tumor classification for the four patients with detected pathogenic germline variants

Ultimately, the reclassification rate for patients histopathologically diagnosed with ependymoma and with available tumor tissue was 7.7% (3/39). Initially, tumor methylation class prediction mandated amendment of the registered diagnosis to a non-ependymoma entity for four patients (Figs. 2 and 3). Two patients with histopathologically diagnosed WHO grade 3 ependymomas located in the pons and thalamus, respectively, were both reclassified as *H3K27*-mutant or *EZHIP* expressing diffuse midline gliomas (DMG_H3K27). Another tumor, extending through the aqueduct from the fourth ventricle also registered as ependymoma in the DCCR, was reclassified as a rosette-forming glioneuronal tumor (RGNT) based on DNA methylation profiling. Of note, the original histopathology report of this tumor revealed a discussion of several differential diagnoses. Finally, an ependymoblastoma incorrectly coded as ependymoma in the registry was specified as a C19mc-altered embryonal tumor with multilayered rosettes (ETMR). All reclassifications were supported by subsequent review of the original histopathology reports by a senior pediatric neuropathologist. For one of the molecularly classified ependymoma patients, a chart review revealed a previous alteration of the initial histopathological diagnosis of atypical glioblastoma to ependymoma based on clinical DNA methylation profiling (Fig. 2).

### Germline DNA sequencing

Tissue for germline DNA sequencing was available for 86% (37/43; new or prospectively collected blood samples n = 28, archived blood samples n = 6, normal brain tissue n = 3) of which 34 retained ependymoma status following molecular tumor classification. The six patients not undergoing germline sequencing were all deceased, part of the retrospective cohort, and without available archived blood samples or dissectible healthy brain tissue in the FFPE tumor samples.

### Cancer panel analysis findings

Nine pathogenic variants (eight SNVs, one SV) in nine patients were detected across the 457 cancer panel genes. Five heterozygous loss-of-function variants in the recessive genes *FANCM, ERCC3,* and *SBDS*, along with relatively common risk allele variants in *CHEK2* and *BRIP1*, were considered unrelated to ependymoma, but are further detailed in Additional file 2: Table S6.

Two of the four pathogenic variants at first assumed to be related to ependymoma were found in children where the histopathological diagnosis was subsequently altered following tumor DNA methylation profiling (Fig. 3). This included an *NF1* nonsense variant (p.Arg1947Ter [c.5839C > T]) in a patient with a molecularly confirmed RGNT and a *TP53* missense variant (p.Arg273Cys [c.817C > T]) in a child with a thalamic DMG_H3K27. Thus, the likelihood of diagnostic reclassification by DNA methylation profiling to a non-ependymoma tumor entity was significantly higher for children with detected pathogenic germline variants (2/4 vs. 0/29, Fisher’s exact test, *p* = 0.011, analysis limited to patients with ependymoma confirmed as initial histopathology diagnosis and both available tumor and germline tissue, n = 33, Additional file 2: Table S4).

A causative 364 bp *NF2* deletion (chr22:30067648–30068012; p.Met334_Leu374del [c.1000-167_1122 + 75del]) was detected in a young child diagnosed with a WHO grade 2 ependymoma (methylation class spinal ependymoma (SP-EPN)) located at the cervicomedullary junction. The patient was initially treated with partial surgical resection followed by focal radiation and adjuvant chemotherapy, after which a minor contrast-enhancing tumor remnant has remained stable for more than 10 years. During follow-up, the patient developed bilateral vestibular schwannomas. Despite a family history with one third generation and several fourth generation relatives with clinically diagnosed neurofibromatosis type-2, the diagnosis had not been suspected until the patient debuted with ependymoma.

Finally, a pathogenic nonsense variant in *LZTR1* (p.Gln762Ter [c.2284C > T]), a gene not formerly linked with ependymoma, was detected in an otherwise healthy child diagnosed with a WHO grade 3 ependymoma (methylation class posterior fossa group A (PF-EPN-A) ependymoma, subclass 1c), located in and around the foramen of Luschka. Of note, the only other *LZTR1* variant observed in our cohort was a VUS (p.Asp703Asn [c.2107G > A]) in another child diagnosed with the same molecular ependymoma subclass (PF-EPN-A1c) in the same location.

No pathogenic variants were detected in the supplementary panel of 67 ependymoma related genes.

### Constrained gene analysis findings

Sixteen pLoF variants (11 SNVs and five SVs) were observed in the same number of constrained genes in 12 patients. Both pLoF variants already known to cause ependymoma (in *NF2* and *NF1*), were rediscovered. However, the nonsense *NF1* variant was found in a patient for whom DNA methylation profiling amended the ependymoma diagnosis to RGNT (Additional file 2: Tables S3 and S5).

Following molecular reclassification, 14 constrained gene pLoF variants remained, and were located in the following genes (ordered according to rising LOEUF scores); *CHD6*, *NF2*, *COL1A1*, *FGD5*, *BRWD1*, *UHRF2*, *ZNFX1*, *FOXO3*, *CDC42BPA*, *DHX37*, *DNAJC2*, *TRIM67*, *ZMYM2*, *VPS4A*. No significant enrichments were detected using the String Database v.11 [30]. However, all but one (*COL1A1*) are expressed in normal brain tissue [31]. Interestingly, 6/7 of the constrained genes in which pLoF variants were found in patients with posterior fossa ependymoma show particularly high expression levels in cerebellar tissue (*UHRF2, FOXO3, CDC42BPA, ZMYM2, CHD6* and *DNAJC2*) [32].

Other than a 3.4-fold enrichment of non-membrane-bounded organelles (false discovery rate 3.56e-3) the GO PANTHER Cellular Component Overrepresentation Test [33] did not reveal any other significant enrichments for the detected constrained genes when compared to all other genes.

## Discussion

In this combined retro- and prospective study, we performed germline WGS/WES and tumor DNA methylation profiling of a population-based cohort diagnosed nationwide over a timespan of 21 years to determine the role of genetic predisposition in childhood ependymoma. Both known cancer genes and genes somatically or epigenetically associated with ependymoma were analyzed for pathogenic germline variants, as were evolutionarily constrained genes. Our findings establish ependymoma as a disease where germline pathogenic variants in known cancer genes only rarely play an underlying role, especially when precise molecular (re)classification is available. We also identify new putative ependymoma predisposition genes. Lastly, we highlight the essential role of including molecular tumor classification in ependymoma studies and the feasibility of using archived tumor samples for this purpose.

### Pathogenic variants detected in known cancer genes

Of the 37 patients undergoing germline WGS, 11% (4/37) were found to harbor pathogenic variants in the cancer panel genes (*NF1*, *NF2, TP53,* and *LZTR1)*). By comparison, both the carrier frequency and the genes involved were similar to the findings of Zhang et al. from their pediatric pan-cancer germline study (n = 1120) which included 67 ependymoma patients (4/67 (6%), *NF1*, *NF2*, *TP53*) [10]. However, in our cohort, tumor DNA methylation profiling reclassified two of the patients with pathogenic germline variants in *NF1* and *TP53* to tumor types other than ependymoma. Consequently, only two pathogenic germline variants were detected among children with molecularly confirmed ependymoma (2/34, 5.9%). In this context, it is worth noting that the reclassification rate in our study (7.7%) is comparable to that reported by Capper et al. [26]. In their prospective cohort of 101 histopathologically diagnosed ependymoma samples, 6.0% (6/101) were reclassified based on tumor DNA methylation profiling to a non-ependymoma entity, including neuroepithelial tumors and two DMGs, similarly to our cohort.

As of this writing, ten large (n > 100) pediatric pan-cancer germline sequencing studies including children with ependymoma have been published (Table 2). Combined, these investigations report pathogenic germline variants in 4.7% (9/191) of children with histopathologically diagnosed ependymoma. Following exclusion of a likely benign *TP53* variant (detailed below), three variants likely unrelated to ependymoma (incidental findings from the ACMG v2.0 [34]) and duplicate patients (detailed in Table 2), just 2.9% (5/173) of children with ependymoma are reported to harbor pathogenic germline variants in known cancer genes. Of these, all were in *NF1* (n = 3) or *NF2* (n = 2). This estimate is strikingly similar to our observations, especially when taking into consideration the low frequencies and sample size and the fact that the gene panels used in the majority of the previous studies did not include *LZTR1*.

**Table 2:** Overview of large (> 100 cases) pan-childhood cancer germline sequencing studies with reported findings for ependymoma

Author, jr	Year	Patients w/pathogenic CPS gene variants (n/total (%))			Comments
		Full childhood cancer cohort	CNS subcohort	Ependymoma subcohort	
Zhang, J (NEJM)	2015	95/1120 (8.5%)	21/245 (8.6%)	4/67 (6.0%)	*NF1* (n = 2), *NF2* (n = 1) and *TP53* (n = 1). The latter has later been assessed as likely benign. Limited to intracranial ependymoma
Parsons, DW (JAMA Onc)	2016	13/150 (8.7%)	2/56 (3.6%)	0/9 (0.0%)	
Oberg, JA (Genome Med)	2016	18/101 (17.8%)	5/17 (29.4%)	2/3 (66.7%)	ACMG secondary findings in *BRCA1* (n = 1) and *VHL (n* = *1)*
Gröbner, SN (Nature)	2018	69/914 (7.6%)	39/542 (7.2%)	0/59 (0.0%)	14 cases are overlapping with Zhang et al. (incl. the patient w the reported *TP53* variant). Limited to intracranial ependymoma
Wong, N (Nature Med)	2020	40/247 (16.2%)	17/92 (18.5%)	0/8 (0.0%)	
Byrjaldsen, A (PLoS Gen)	2020	29/198 (14.7%)	3/44 (6.8%)	0/4 (0.0%)	Ependymoma cases (n = 4) overlap with the current study
Fiala, EM (Nature Can)	2021	138/751 (18.4%)	30/143 (21.0%)	3/14 (21.4%)	*NF1* (n = 1), *NF2* (n = 1) and an ACMG secondary finding in *FANCA* (n = 1)
Newmann, S (Cancer Discovery)	2021	55/300 (18.3%)	19/97 (19.6%)	0/11 (0.0%)	
Stedingk, KV (Sci rep)	2021	30/790 (3.8%)	8/149 (5.4%)	0/14 (0.0%)	Limited to SNV analysis
Wagener, R (EJHG)	2021	11/160 (6.9%)	3/32 (9.4%)	0/2 (0.0%)	
**Total**		**509/4833 (10.5%)**	**147/1425 (10.3%)**	**9/191 (4.7%)**	
*Adjusted total for ependymoma*				*5/173 (2.9%)*	Excl. ACMG secondary findings, 14 cases overlapping in Zhang et al./Gröbner et al. and the four cases reported by Byrjalsen et al. also in the current cohort
*Our study*				*2/34 (5.9%)*	Restricted to molecularly confirmed ependymoma
**Current best estimate**				**7/207 (3.4%)**	

ACMG, American College of Medical Geneticists

### Neurofibromatosis type-2 predisposes both to intraspinal and -cranial childhood ependymoma

The association between neurofibromatosis type-2 and spinal ependymoma is well established [35] and somatic *NF2* variants are recurrently altered in ependymomas with intraspinal location [36]. Yet, several cases of intracranial ependymoma (especially located to the cervicomedullary junction) have been reported in children and young adults with neurofibromatosis type-2 [37–41]. Combined with our findings of a cervicomedullary located ependymoma in a child with a pathogenic germline *NF2* variant, there is mounting evidence that germline *NF2*-related ependymomas may be located intracranially, as well as intraspinally. While the former will often represent SP-EPN located in or around the cervicomedullary junction, cases of PF-EPN-B ependymoma have also been reported [37].

Still, pathogenic germline *NF2* variants are relatively rare in the overall pediatric ependymoma population and thus explain only a minority of cases: Among the 173 children with ependymoma included in the reviewed pan-childhood cancer germline sequencing studies [10–19], only two patients (1.2%) were reported to harbor pathogenic *NF2* alterations [10, 16] (Table 2), for whom neither tumor location nor molecular subclass were described.

### Questioning Li-Fraumeni Syndrome’s association with (molecularly classified) ependymoma

Both somatic and germline *TP53* variants have been reported in other pediatric CNS tumors, yet such alterations are extremely rare in ependymoma tumor tissue [42]. Of all the children with ependymoma included in the aforementioned germline predisposition investigations, only one patient (0.6%, 1/173) was found to carry a *TP53* variant characterized as pathogenic [10]. The variant (NM_000546:p.Tyr107His, c.319T > C), which was detected in a 10-year-old girl with an infratentorial ependymoma, has later been classified as benign in ClinVar [43] and was not reported as pathogenic by Gröbner et al., who included the same patient in their subsequent study [13]. Furthermore, the variant has been found in 0.1% of healthy adults that self-identified as African/African American [24]. Apart from the 173 children with ependymoma reviewed above, five cases of children with ependymoma and pathogenic germline *TP53* variants have been reported in the literature [44–46]. Of note, molecular tumor classification was not performed in any of these cases. Were it not for DNA methylation profiling-based reclassification to DMG, the erroneous ependymoma phenotype in our cohort would have been reported as associated with the germline *TP53* variant. This underscores the importance of molecular classification of ependymal tumors.

### Pathogenic NF1 germline variants also appear to play a role in childhood ependymoma

Pathogenic *NF1* germline variants are extremely rare among children with ependymoma. No such variants were detected among the 34 children with molecularly classified ependymoma following diagnostic revision to RGNT for the child with a nonsense variant in *NF1*. In comparison, three of the reported 173 germline sequenced children with ependymoma (1.7%) have been found to carry pathogenic *NF1* variants (Table 2). These include two children with intracranial ependymoma reported by Zhang et al. [10] and one 6-year-old child with synchronous schwannoma and CNS ependymoma reported by Fiala et al. [16]. Only two additional cases of children with (clinically) diagnosed neurofibromatosis type-1 and intracranial ependymoma have been reported in the literature [47]. Diagnostic confirmation and tumor molecular subtyping by DNA methylation profiling was not reported for any of these patients. This may have inflated the reported *NF1* carrier rate in patients with ependymoma. This phenomenon is illustrated by the diagnostic revision in both our cohort and others, where histopathologically diagnosed ependymomas were reclassified to pilocytic astrocytomas and neuroepithelial tumors based on DNA methylation profiling [26]. Importantly, both of these tumor types have a much higher rate of germline *NF1* alterations [48, 49].

### LZTR1 might represent a novel putative ependymoma predisposition gene

A likely pathogenic *LZTR1* variant (p.Gln762Ter [c.2284C > T]), undetected among > 125,000 healthy adult in gnomAD [24], was found in a child diagnosed with a fourth ventricle PF-EPN-A1c ependymoma. Pathogenic germline variants in *LZTR1* have not previously been reported in patients with ependymoma. The gene, which is centromeric to *NF2* and *SMARCB1* on chromosome 22q11.21, was recently uncovered as a germline predisposition gene in schwannomatosis [50]. Pathogenic *LZTR1* germline variants have been reported in children with different cancer types, including high-grade glioma [13], but have not been evaluated in the majority of the existing large pan-childhood cancer germline sequencing studies [10, 11, 16–18]. Although monozygosity of 22q has been reported in ~ 40% of RELA-fusion positive supratentorial ependymoma (ST-EPN-RELA) [52], the rarity of pathogenic somatic *NF2* variants in the majority of intracranial ependymoma suggests a different tumor suppressor gene to be located on chromosome 22 [51, 53, 54]. We therefore speculate that pathogenic germline *LZTR1* variants may play a role in tumorigenesis for a limited subset of children with ependymoma, perhaps restricted to the PF-EPN-A1c molecular subtype.

Upon review of *LZTR1* findings in our childhood (non-ependymoma) cancer control cohort, the *LZTR1* missense VUS (p.Asp703Asn [c.2107G > A]) detected in another patient with PF-EPN-A1c was observed in a child with acute myeloid leukemia. Moreover, this variant has been reported in 5/26,128 (0.02%) Swedish individuals reported without serious childhood disease in gnomAD [24].

### Less than 4% of childhood ependymoma is explained by pathogenic variants in known cancer genes

Based on the described meta-analysis, the current best estimate of germline predisposition in childhood ependymoma suggests that 3.4% (7/207) carry a causative pathogenic germline variant, mainly located in *NF2* and *NF1* (Fig. 2). This estimate indicates that germline predisposition is significantly less frequent than what is reported for pediatric brain and spinal cord tumors in general [3] (Fisher’s exact test, 7/207 vs. 147/1425, OR = 0.30 [0.11–0.66], *p* < 0.001). Consequently, one may question the need to perform extensive genetic testing in newly diagnosed children with ependymoma if no family history or other signs or symptoms of neurofibromatosis are present. Of course, the lack of germline findings in the majority of children with ependymoma may reflect limitations in our current knowledge of genetics. Or, perhaps more likely, other biological mechanisms including epigenetic dysregulation, which has been suggested as the main driver for the largest molecular subgroup, PF-EPN-A [55, 56].

Several factors may, however, have influenced the validity of the combined risk estimate; Opposite to our study, all but one of the germline investigations listed in Table 2 did not report molecular tumor classification [12]. Moreover, their lack of population-based study design may have introduced selection bias. As illustrated by the pathogenic *NF2* deletion detected in our cohort, limiting bioinformatic analyses solely to SNVs, as done in one of the reviewed sequencing studies [18], may miss pathogenic alterations. Also affecting the generalizability of the combined estimate is the fact that the two cohorts contributing 65% (112/173) of the total ependymoma sample size were limited to intracranial ependymoma, likely resulting in underreporting of *NF2*-associated cases [10, 13].

### Constrained gene analysis may explain additional genetic risk

Focusing on genes exhibiting evolutionary intolerance of inactivating alterations has recently emerged as a novel approach of investigating genetic predisposition to any state that limits reproduction, such as fatal childhood diseases [24]. We have previously detailed how a constrained gene approach may be useful in investigations of genetic predisposition to childhood (CNS) malignancies [21].

Constrained gene analysis of children with molecularly confirmed ependymoma rediscovered the *NF2* deletion detected in our cancer gene panel analysis. Apart from *NF2*, none of the 14 constrained genes in which pLoF variants were detected have previously been linked with ependymoma. Interestingly, several are suggested to have tumor suppressor roles (*FOXO3* [57], *TRIM67* [58], *UHRF2* [59, 60], *CHD6* [61]).

As no single gene was found to harbor pLoF variants in more than one patient, further research of the concept is needed before a common or broader role for constrained genes in ependymoma predisposition can be ascertained. In our cohort, the lack of consistent constrained gene findings likely reflects the limited sample size and its subtype heterogeneity, or, alternatively, the growing notion that PF-EPN-A is an epigenetically driven disease. In this context, it is worth mentioning that two of the constrained genes, in which pLoF variants were detected in children with PF-EPN-A, affect epigenetic gene expression control (*UHRF2* [60, 62] and *DNAJC2* [63]). As neither the detected constrained genes nor *LZTR1* have been analyzed in the majority of the aforementioned pediatric pan-cancer germline sequencing studies, their inclusion in future larger ependymoma cohorts will be important to confidently suggest any disease-related roles and indication for further study.

### Strengths and limitations

Key strengths of this study include its population-based design, high inclusion rates and molecular tumor classification based on DNA methylation profiling. Moreover, our germline WGS-based SNV and SV and WES SNV analysis included not only 390 known cancer genes, but also 67 other genes with implied roles in ependymoma tumorigenesis and constrained gene analysis. The comprehensive literature review-based meta-analysis further strengthens the value of our investigation.

However, even with a nationwide inclusion period of more than 20 years, our sample size limits generalizability of the observed carrier frequencies. Tumor and germline tissue were unavailable for four and six patients, respectively. Finally, the use of a non-ependymoma childhood cancer control cohort in the filtering of germline variants might have affected variant filtration in a conservative direction. Optimally, an equal or larger control cohort of representative and ethnically comparable whole-genome sequenced children would have been available.

### In summary

This population-based germline sequencing study of childhood ependymoma, including constrained gene analysis, establishes that genetic predisposition plays a role for less than 4% of patients. This is significantly lower than for pediatric CNS tumors in general. Moreover, we show that pathogenic germline variants in children with ependymoma are virtually restricted to *NF2* and *NF1*. Our results emphasize the importance of molecular tumor classification, as the likelihood of diagnostic reclassification to a non-ependymoma tumor was significantly higher for children with detected pathogenic germline variants. We therefore advocate diagnostic reconsideration in children with non-molecularly classified ependymoma with cancer predisposition syndromes other than neurofibromatosis type-2. In addition, we present *LZTR1* as a novel putative ependymoma predisposition gene.

## Supplementary Information

**Additional file 1**. Redefining germline predisposition in children with molecularly characterized ependymoma: a population-based 20-year cohort. **Figure S1**. Flowchart illustrating the filtering of single nucleotide germline variants in 37 children with histopathologically diagnosed ependymoma. **Figure S2**. Flowchart illustrating the filtering of structural germline variants in 37 children with histopathologically diagnosed ependymoma. **Figure S3**. Overview of the inclusion process and germline tissue availability.

**Additional file 2: Table S1**. Overview of the 390 genes associated with cancer included in the panel analysis. **Table S2**. Genes reported with potential germline/somatic role in ependymoma. **Table S3**. Constrained genes manual curation results. **Table S4**. Cohort overview: clinical, histopathological and molecular data. **Table S5**. Overview of pathogenic variants in known cancer associated genes and pLoF variants in evolutionarily constrained genes by molecular tumor type. **Table S6**. Five heterozygous loss-of-function variants in recessive genes considered unrelated to ependymoma.

## Data Availability

All data produced in the present work are contained in the manuscript with the exception of genetic sequencing data. Danish legal regulation does not permit uploading of raw sequencing data. Selected data may be made available upon reasonable request (dependent on required approvals from relevant scientific ethic boards) to the authors.
