# Supplementary material for: Redefining germline predisposition in children with molecularly characterized ependymoma: a population-based 20-year cohort
Source: Acta Neuropathol Commun. 2022 Aug 25;10:123. doi: 10.1186/s40478-022-01429-1 (PMC9404601; doi:10.1186/s40478-022-01429-1)
Supplement: Supplementary file 1 — Additional file 1. Redefining germline predisposition in children with molecularly characterized ependymoma: a population-based 20-year cohort. Figure S1. Flowchart illustrating the filtering of single nucleotide germline variants in 37 children with histopathologically diagnosed ependymoma. Figure S2. Flowchart illustrating the filtering of structural germline variants in 37 children with histopathologically diagnosed ependymoma. Figure S3. Overview of the inclusion process and germline tissue availability. [file 40478_2022_1429_MOESM1_ESM.docx]

**Additional file 1: Redefining germline predisposition in children with molecularly characterized ependymoma: a population-based 20-year cohort**

**ADDITIONAL FILE METHODS**

*Germline whole-genome and -exome sequencing*

Germline WES of healthy brain tissue was performed for deceased patients with no other tissue samples available. A senior neuropathologist reviewed archived formalin-fixed and paraffin-embedded (FFPE) tumor samples for presence of normal brain tissue, and a section of healthy tissue was dissected with the greatest possible distance from deceased tissue. Next, DNA was purified using QIAamp DNA FFPE Tissue Kit (QIAGEN, Germany) according to manufacturer’s instructions. A minimum of 50ng of DNA was fragmented to an average size of 150bp using the Covaris E220 focused-ultrasonicator. Adaptor ligation was performed using the NEBNext Ultra DNA II Library prep (New England BioLabs, USA) protocol for low input samples on a Sciclone G3 robot (Perkin Elmer, USA). Exome capture was done using SureSelectXT Clinical Research Exome V2 kit (Agilent, USA) followed by sequencing on Novaseq 6000 (Illumina, USA). Exomes were sequenced as 2 x 150 bp paired-end reads to an average median coverage of 60X.

Reads were subsequently mapped to the hg19 reference genome sequence (GRCh37.p13; RefSeq assembly accession GCF_000001405.25) using the DNAseq pipeline (Sentieon, USA) or GATK version 3.8.

For WGS data, structural variants (SVs) were called using a combination of CNVnator (0.3.3), Manta (1.4), Delly2 (0.8.1) and CNV kit (0.9.6) with post-processing in R (3.6.1). Variant annotation was performed using VarSeq software (version 2.2.3, Golden Helix, USA). The employed WGS protocol has previously been described in further detail[^1^](https://www.zotero.org/google-docs/?QWwKKn).

*Variant filtering*

Annotated, raw single nucleotide variant (SNV) and structural variant (SV) files were loaded into R. Variants were then filtered as illustrated in Additional file 1: Figures S1 and S2. For SNVs, only variants passing quality control (QC) parameters [allele count=2, VAF >0.3 and <0.70, coverage >15X, strand bias <10, indel size <10] and designated non-synonymous were included. For SVs, variants present in a Danish pediatric clinical non-cancer cohort (n=210, critically ill children under suspicion of metabolic disease or epileptic encephalopathy) were filtered out. Next, variants were filtered for rarity (population frequency of <1% in gnomAD v2.1[^2^](https://www.zotero.org/google-docs/?AE4fyV) and/or observed <3 times among 586 children with cancers other than ependymoma) and subsetted to the relevant variants in patients registered with ependymoma. SV analysis was restricted to deletions.

*Constrained gene analysis*

The resulting constrained gene variants underwent manual curation for veracity and possible pathogenicity by a combination of visual analysis in the Integrated Genome Viewer, comparison with ClinVar for variant classification[^3^](https://www.zotero.org/google-docs/?c9x0Xf), and population frequencies in gnomAD[^2^](https://www.zotero.org/google-docs/?1zxm9y). Subsequently, the passing variants were assessed for possible associations with cancer processes by scientific literature review, as well as analyses using the Gene Ontology (GO) knowledgebase[^4^](https://www.zotero.org/google-docs/?3utVJt) and the String Database (String-dB) v.11[^5^](https://www.zotero.org/google-docs/?2VTTou)**.** Manual curation results are detailed in Additional file 2: Table S3.

*Molecular tumor classification*

Unprocessed IDAT files were retrospectively collected for patients with existing clinical tumor methylation profiles. For all others, available FFPE tumor samples were retrieved from pathology biobanks nationwide. For patients without obtainable FFPE tumor samples, freshly frozen (FF) tumor tissue was retrieved if available. Methylation profiling was performed using the Infinium MethylationEPIC BeadChip Kit (Illumina, USA) according to the manufacturer’s instructions. Resulting unprocessed IDAT files were uploaded to and processed by a publicly available classifier tool to predict tumor methylation class[^6^](https://www.zotero.org/google-docs/?BkhIAU).

Methylation profiling for patients without existing clinical profiles with available FFPE or FF tissue was conducted using the Infinium MethylationEPIC BeadChip Kit (Illumina, USA) according to the manufacturer’s instructions. Resulting unprocessed IDAT files were uploaded to and processed by a publicly available classifier tool to predict tumor methylation class[^6^](https://www.zotero.org/google-docs/?8DK0mU). For assignment of tumor methylation class a calibrated score of >0.9 in either the brain_classifier_v11b4 (v.3.1) or brain_classifier_v12.3 (v1.1) was required. For methylation subclass, a score of >0.5 was accepted. Copy number variation (CNV) plots provided by the classifier tool were registered.

**ADDITIONAL FILE RESULTS**

Existing clinical tumor DNA methylation profiles were available for 18 participants, of whom eight were prospectively included patients, reflecting a change towards using tumor methylation profiling routinely over recent years. Of the 25 patients without clinical methylation profiles, biobanked FFPE tumor tissue was retrievable for 88% (22/25). FF tumor tissue was available for just one of the remaining three patients. Thus, either existing clinical methylation profiles or archived tumor tissue samples were available for 95% (41/43). The two patients with neither clinical methylation profiles nor archived tumor tissue samples available, were both in the retrospective cohort and diagnosed prior to 2009. Two of the 22 archived FFPE samples failed to meet the internal QC parameters of the classifier tool, possibly due to low DNA amount or low tumor cell content. Mean storage time did not differ significantly between archived FFPE samples passing (n=20) and failing (n=2) the classifier tool QC (15.1 vs 16.3 years, Mann Whitney U-test p=0.866). Clinical methylation profiles for four of the retrospective patients had been established using the Illumina Human Methylation 450 BeadChip array, while all others were attained through the Illumina Infinium MethylationEPIC BeadChip Kit.

Consequently, WGS of leukocyte DNA or healthy brain tissue WES was performed for 32 and two patients, respectively, for whom methylation profiling did not amend the ependymoma diagnosis.

**ADDITIONAL FILE DISCUSSION**

Adding to the challenge of estimating the true contribution of genetic predisposition is the lack of other systematic germline sequencing investigations specific to childhood ependymoma, and the fact that a substantial part of the existing related literature is comprised of case reports and syndrome-specific cohorts (e.g. Li Fraumeni and neurofibromatosis type-1), which are prone to ascertainment bias. *TRIM67*, in which a damaging variant was detected in a young child with ST-EPN-ZFTA, has recently been described as a tumor suppressor gene which directly inhibits p53 degradation by its ubiquitin ligase MDM2[^7^](https://www.zotero.org/google-docs/?yUBFc7). Of note, increased *MDM2* expression and *MDM2* amplification has been shown in 100% and 33% of examined supratentorial ependymoma samples, respectively, and suggested as a molecular driver in ependymoma[^8^](https://www.zotero.org/google-docs/?t4Iqa4).

**ADDITIONAL FILE FIGURES**

**Additional file 1: Figure S1. Flowchart illustrating the filtering of single nucleotide germline variants in 37 children with histopathologically diagnosed ependymoma**


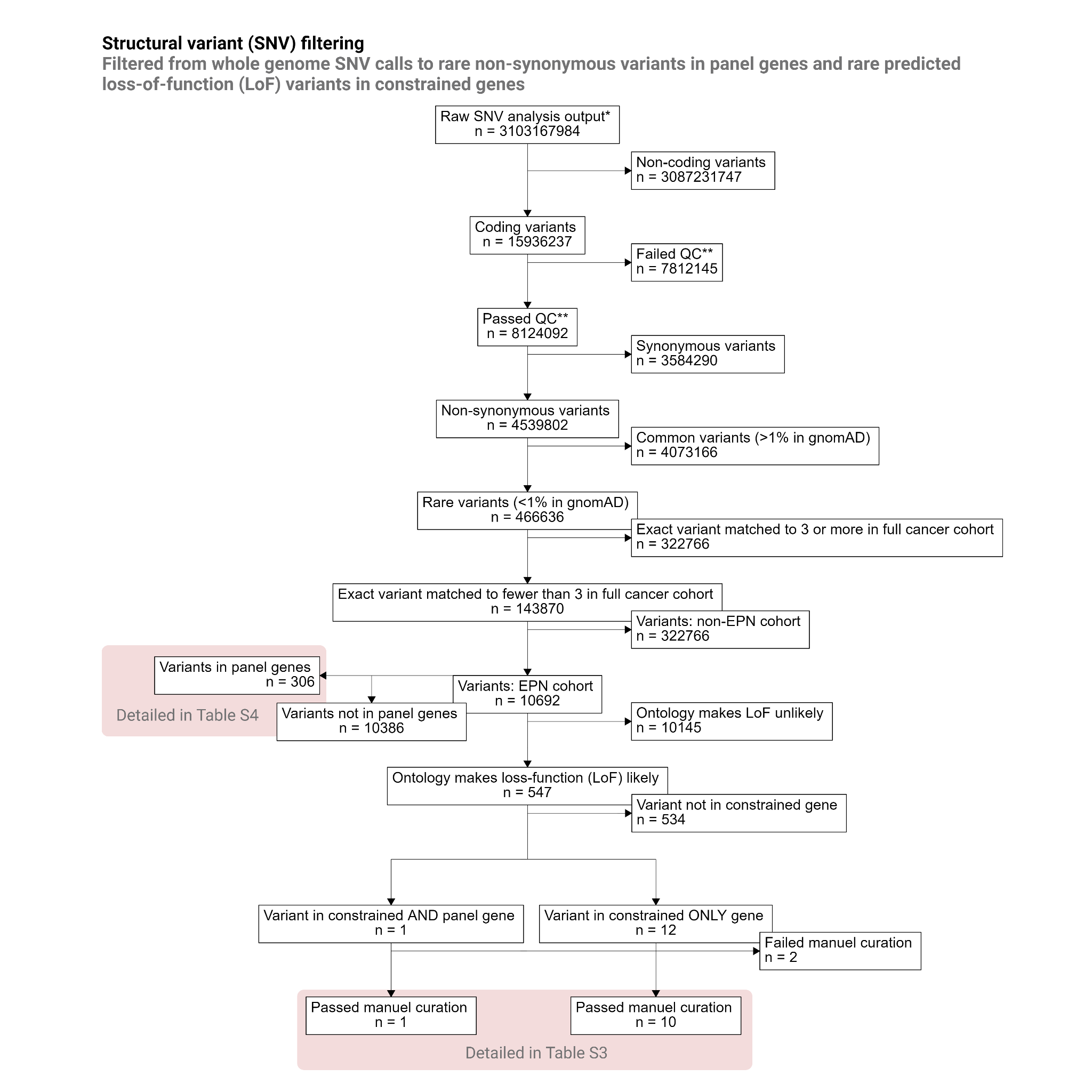


**Additional file 1: Figure S2. Flowchart illustrating the filtering of structural germline variants in 37 children with histopathologically diagnosed ependymoma**


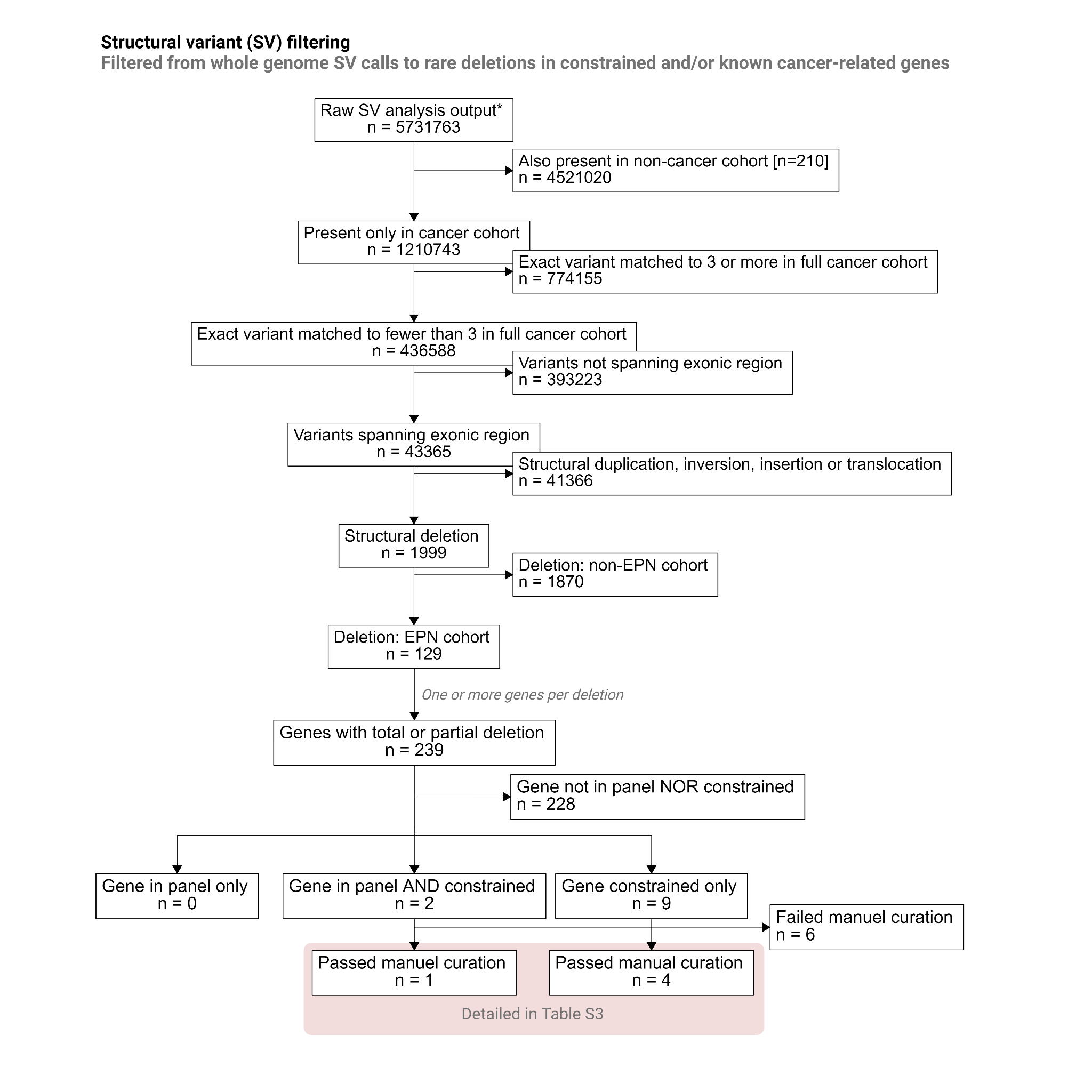


**Additional file 1: Figure S3. Overview of the inclusion process and germline tissue availability**

**
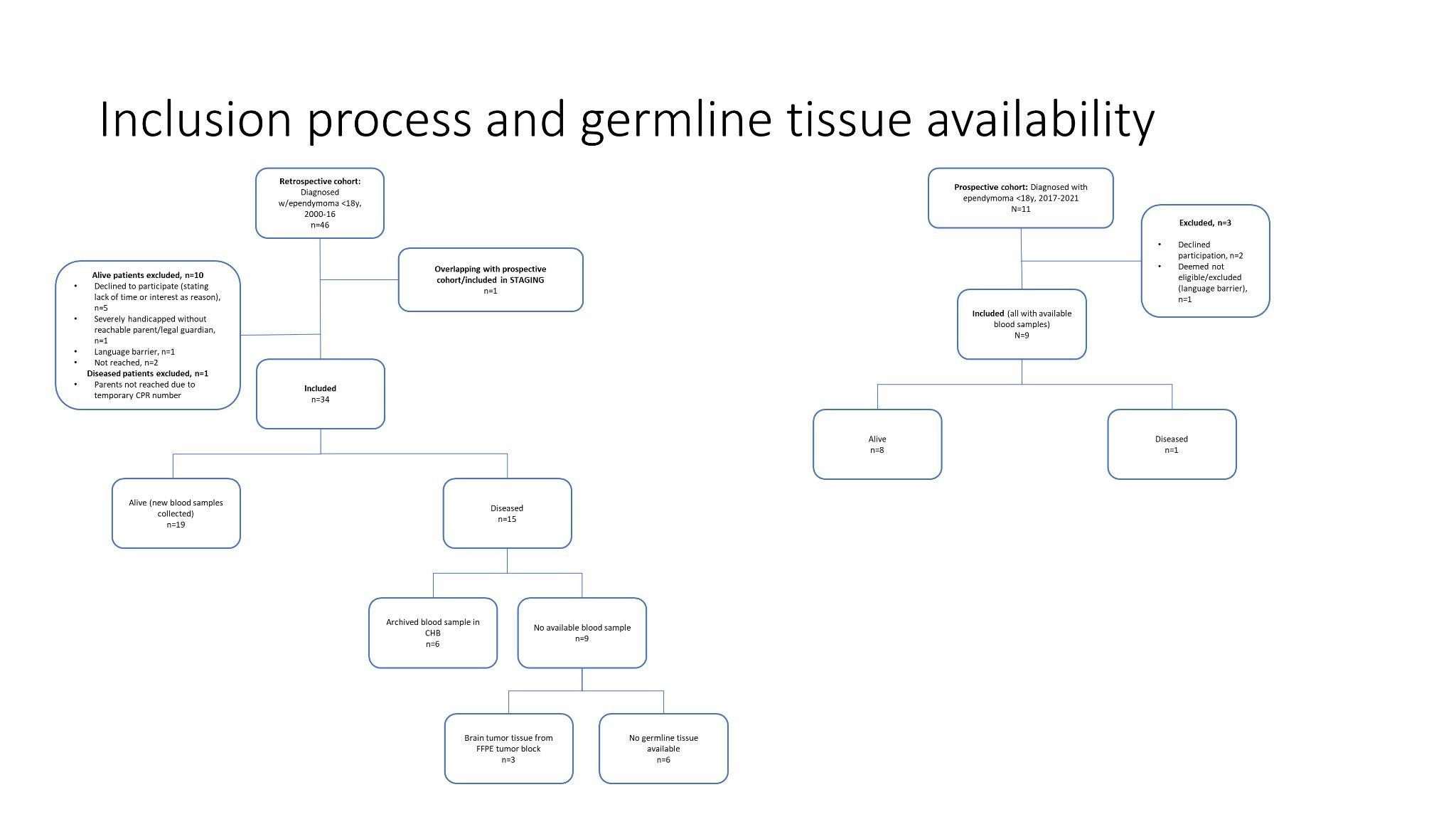
**

**REFERENCES FOR THE ADDITIONAL FILE DATA:**

[1. Byrjalsen A, Hansen TVO, Stoltze UK, et al. Nationwide germline whole genome sequencing of 198 consecutive pediatric cancer patients reveals a high frequency of cancer prone syndromes. *PLoS Genet*. Published online 2020. doi:10.1371/JOURNAL.PGEN.1009231](https://www.zotero.org/google-docs/?2K0YEF)

[2. Karczewski KJ, Francioli LC, Tiao G, et al. The mutational constraint spectrum quantified from variation in 141,456 humans. *Nature*. 2020;581(7809):434-443. doi:10.1038/s41586-020-2308-7](https://www.zotero.org/google-docs/?2K0YEF)

[3. Landrum MJ, Lee JM, Benson M, et al. ClinVar: improving access to variant interpretations and supporting evidence. *Nucleic Acids Res*. 2018;46(D1):D1062-D1067. doi:10.1093/nar/gkx1153](https://www.zotero.org/google-docs/?2K0YEF)

[4. Gene Ontology Consortium. The Gene Ontology resource: enriching a GOld mine. *Nucleic Acids Res*. 2021;49(D1):D325-D334. doi:10.1093/nar/gkaa1113](https://www.zotero.org/google-docs/?2K0YEF)

[5. Szklarczyk D, Gable AL, Lyon D, et al. STRING v11: protein-protein association networks with increased coverage, supporting functional discovery in genome-wide experimental datasets. *Nucleic Acids Res*. 2019;47(D1):D607-D613. doi:10.1093/nar/gky1131](https://www.zotero.org/google-docs/?2K0YEF)

[6. Capper D, Jones DTW, Sill M, et al. DNA methylation-based classification of central nervous system tumours. *Nature*. Published online 2018. doi:10.1038/nature26000](https://www.zotero.org/google-docs/?2K0YEF)

[7. Wang S, Zhang Y, Huang J, et al. TRIM67 Activates p53 to Suppress Colorectal Cancer Initiation and Progression. *Cancer Res*. 2019;79(16):4086-4098. doi:10.1158/0008-5472.CAN-18-3614](https://www.zotero.org/google-docs/?2K0YEF)

[8. Suzuki SO, Iwaki T. Amplification and Overexpression of mdm2 Gene in Ependymomas. *Mod Pathol*. 2000;13(5):548-553. doi:10.1038/modpathol.3880095](https://www.zotero.org/google-docs/?2K0YEF)
